# Rapid thermal annealing and crystallization mechanisms study of silicon nanocrystal in silicon carbide matrix

**DOI:** 10.1186/1556-276X-6-129

**Published:** 2011-02-10

**Authors:** Zhenyu Wan, Shujuan Huang, Martin A Green, Gavin Conibeer

**Affiliations:** 1 ARC Photovoltaics Centre of Excellence, University of New South Wales (UNSW), Sydney, Australia

## Abstract

In this paper, a positive effect of rapid thermal annealing (RTA) technique has been researched and compared with conventional furnace annealing for Si nanocrystalline in silicon carbide (SiC) matrix system. Amorphous Si-rich SiC layer has been deposited by co-sputtering in different Si concentrations (50 to approximately 80 v%). Si nanocrystals (Si-NC) containing different grain sizes have been fabricated within the SiC matrix under two different annealing conditions: furnace annealing and RTA both at 1,100°C. HRTEM image clearly reveals both Si and SiC-NC formed in the films. Much better "degree of crystallization" of Si-NC can be achieved in RTA than furnace annealing from the research of GIXRD and Raman analysis, especially in high-Si-concentration situation. Differences from the two annealing procedures and the crystallization mechanism have been discussed based on the experimental results.

## Introduction

Shockly and Queisser [1] have calculated the upper theoretical efficiency limitation for on p-n junction silicon solar cell as 30%. In order to further obtain a higher efficiency, multi-junction solar cells with different materials have been designed and fabricated [2]. However, to create different band gap solar cell layers, expensive and perhaps toxic materials have to be involved and this is assumed to be the main obstacle for the wide use of multi-junction solar cell. As a result, in recent years, the theory of "all silicon multi-junction solar cell" has been developed [3,4], and silicon nanocrystals (Si-NCs) in various dielectric materials study have gained researchers' interests in all silicon multi-junction solar cell applications [5]. Due to quantum size effect, three-dimensional quantum-confined silicon dots have been proven to be able to tune the bandgap in a wide range by controlling the dot size. The bandgap of each cell layer can be adjusted by the wavelength of different light spectrum and all silicon multi-junction solar cells with high efficiency can be well expected.

Many research efforts have been allocated in looking for a better dielectric material as a matrix to embed the Si-NC. Comparing the band gap with different materials such as silicon dioxide (approximately 8.9 eV) and silicon nitride (approximately 4.3 eV), the band gap of silicon carbide (approximately 2.4 eV) is the lowest [5]. The small SiC bandgap increases the electron tunnelling probability. Increased carrier transportation performance and greater current can be expected from these multi-junction solar cells. Kurokawa et al. and M. Künle et al. [6,7] have reported the fabrication of good quality Si-NC in SiC matrix film by plasma-enhanced chemical vapor deposition (PECVD) system. However, the main disadvantages of PECVD deposition are extremely time consuming in superlattice structure and in toxic, explosive, and expensive gases involved, such as silane (SiH_4_), monomethylsilane (MMS), methane (CH_4_), and hydrogen (H_2_) etc. In our group, Si-NCs in a SiC matrix deposited by a sputtering process have been intensively investigated in order to overcome the disadvantages listed above.

In our previous research, Si-NCs are fabricated by post-deposition annealing of Si-rich SiC (SRC) layer in a nitrogen furnace for a long time (more than 1 h) [8,9]. Both Si and SiC NC have been clearly observed in x-ray diffraction (XRD) and transmission electron microscopy (TEM) measurements when annealing temperature rise above 900°C. After annealing, SiC-NCs in beta phase (β-SiC) as well as amorphous Si are found surrounding the Si-NC. Rapid thermal annealing (RTA) has been considered as a primary annealing technique in semiconductor industry because of the low energy cost and better crystallization result [10,11] In nanocrystalline system, better crystallization has also been reported in RTA because heating of the structure is caused by light directly absorbed in the layers [12]. In this paper, we compare two annealing techniques: conventional furnace annealing and RTA upon Si and SiC nanocrystalline system, and subsequently research the differences of structural characterization. By investigating the crystallization differences, we try to explain the crystallization mechanism of Si and SiC-NC.

### Experimental details

The SRC films are deposited by magnetron co-sputtering a Si and a SiC target at room temperature using a multi-target sputtering machine (AJA International, ATC-2200, North Scituate, MA, USA). Radio frequency (RF, 13.56 MHz) power supplies are connected to the targets. The Si concentration in the SRC films is controlled by adjusting the RF supply power connected to the Si target. The base pressure of the main chamber of deposition was 8.0 × 10^-^^7 ^Torr and the working pressure is 2.0 × 10^-^^3 ^Torr. Table 1 includes the sample details reported in this paper.

**Table 1:** Sample names and deposition conditions

Sample name	Silicon-rich concentration (volume percentage v%)	Sample structure/thickness (nm)
SRC80	80	Single layer/approximately 600
SRC70	70	Single layer/approximately 600
SRC60	60	Single layer/approximately 600
SRC50	50	Single layer/approximately 600
SiC	0	Single layer/approximately 600

After deposition, either furnace or RTA annealing is carried out for the purpose of Si precipitation from the matrix. The furnace annealing is processed in nitrogen (N_2_) ambient at 1,100°C for 1 h with 40 min ramping-up time from 500°C to 1,100°C. The RTA annealing is also processed in N_2 _ambient at 1,100°C, but with a very short ramping time of 45 s in the same temperature range and much shorter annealing time of 2 min. A detailed temperature ramping profile is listed in Table 2.

**Table 2:** Temperature ramping profile for conventional furnace annealing and RTA

	Room temperature, approximately 500°C	500°C to approximately 900°C	900°C to approximately 1,100°C	1,100°C
Conventional furnace annealing	N/A	25 min	15 min	60 min
RTA	15 min	30 s	15 s	2 min

The structural properties including the nanocrystal size, shape, and phase separation are studied using TEM (Phillips CM200) at 200 kV. The crystalline properties are evaluated by grazing incidence XRD using a Philips's X'Pert Pro material research diffraction system at a voltage of 45 kV and a current of 40 mA, using Cu Kα radiation (*λ *= 1.5418 Å). The glancing angle of the incident x-ray beam is optimised by omega scan and set between 0.2° and 0.4° The nanocrystal size is estimated using the Scherrer equation. Additional structural properties such as phase separation and crystallinity are studied by Raman spectroscopy (Renishaw, RM2000) in backscattering configuration. The power of the Ar ion laser (514 nm) was reduced below 8 mW to avoid local crystallization by laser beam.

## Results and discussion

### TEM study

**Figure 1:**
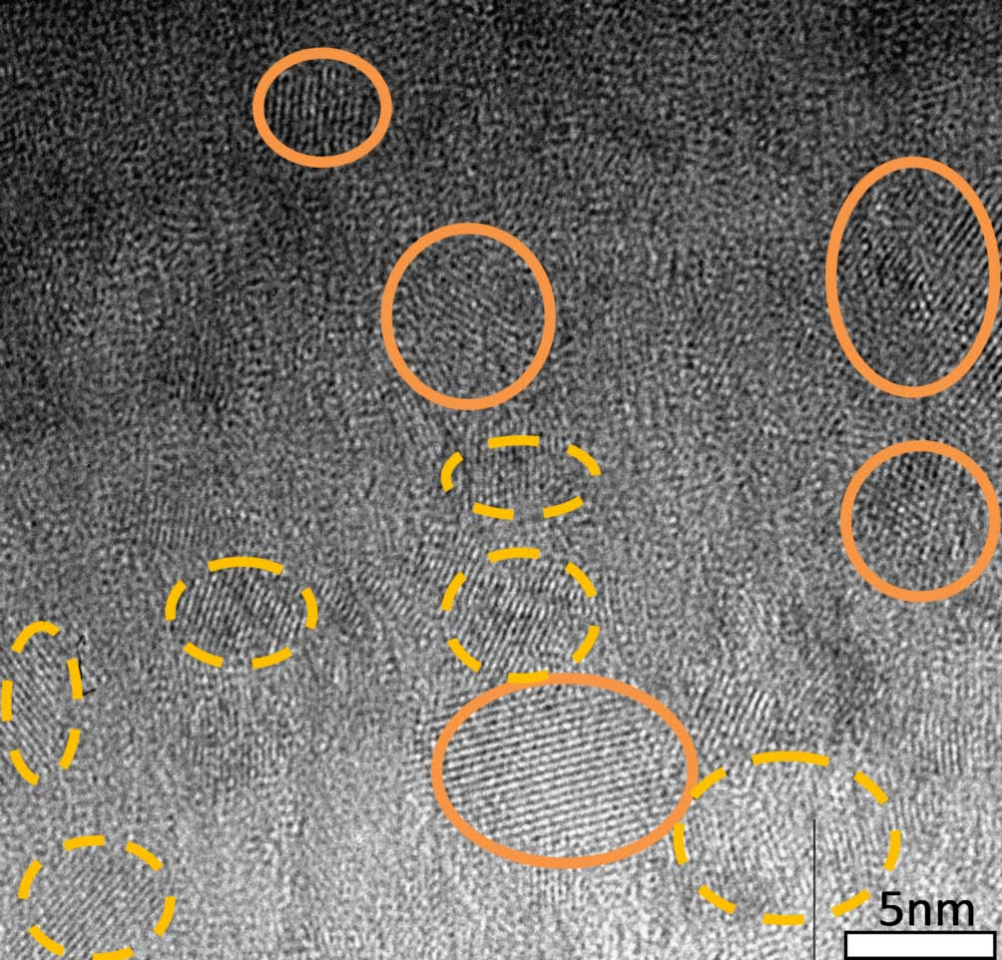
HRTEM plan view of image of SRC50 sample annealed by RTA.

**Figure 2:**
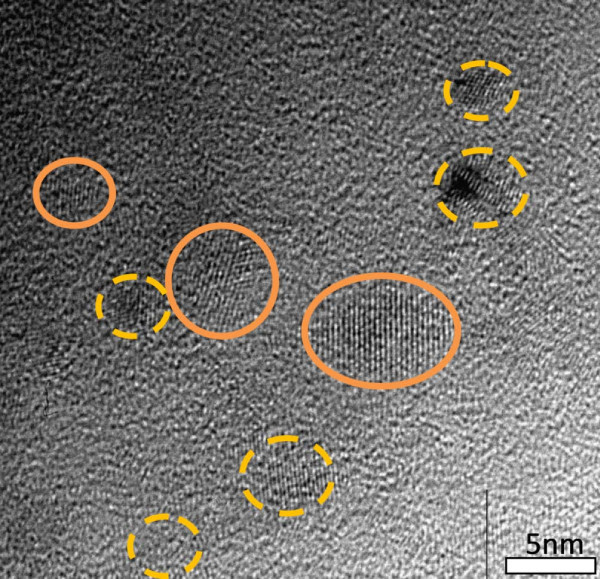
Cross-section TEM image of SRC50 sample annealed by furnace.

Figures 1 and 2 show the plan view TEM images of the sample SRC50 after RTA and furnace annealing. The volume percentage of Si over SiC is 50 v% from RF sputter rates of Si and SiC are calibrated by crystal thickness monitor. Both images clearly reveal the formation of NC. The NC which is circled by solid lines with a fringe spacing 3.1 Å corresponds to Si (111) lattice plane; and the dash-line which is circled with a fringe spacing of 2.5 Å corresponds to the lattice plane of β-SiC (111) [8]. The nanocrystal size and shape are similar in both annealing conditions, with Si size 6-7 nm and SiC size 2-3.5 nm.

### X-ray diffraction investigation

**Figure 3:**
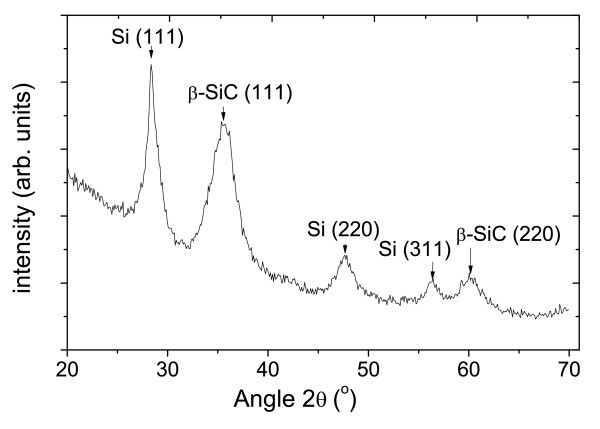
Wide scan XRD curve of the sample SRC60 annealed by furnace.

The crystalline properties of samples annealed by RTA and furnace are studied by XRD. Figure 3 shows a wide scan XRD curve of the sample SRC60 annealed by furnace. The Bragg peaks can be assigned to cubic Si nanocrystal as well as β-SiC nanocrystal, as shown by the indexes in the graph. This suggests the formation of both Si and β-SiC-NC which is consistent to TEM results.

**Figure 4:**
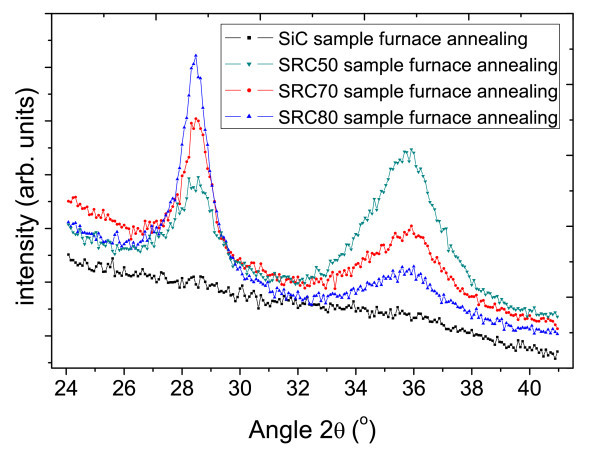
XRD curves of the samples with different Si concentrations after furnace annealing.

Figure 4 compares the XRD spectra of the samples with different Si concentrations after 1,100 C annealing. All the annealed samples show clear Bragg peaks from Si and β-SiC crystallization. In addition, the intensity of Si Bragg peak increases while the SiC peak decreases with the increasing of Si concentration. This phenomenon can be explained by more amorphous silicon (a-Si) is involved in precipitation and crystallization, as a result, higher crystallization volume of crystallized-Si can be achieved. This reason can also be used to explain SiC peaks: when Si concentration increase, SiC concentration decreases, and the volume of SiC crystallinity decreases due to less available a-SiC.

It should be noted that there is no Bragg peak of β-SiC phase detected from a sputtered stoichiometric SiC film, indicating that SiC film does not crystallize under 1,100°C annealing condition itself due to insufficient kinetic energy [13]. That both Si and SiC-NC appear in silicon-rich carbide samples could be due to the Si inducement. Some researchers reported sputtered Si starts to crystallize at 900°C [14]. Si and SiC-NC could be observed after annealing at 900°C in our previous research [8,9]. From these results, we propose that at annealing temperatures of 900°C, the formation of Si-NC [8], act as nuclei for SiC nanocrystal growth. As a result, both Si and SiC diffraction peaks could be observed in silicon-rich carbide samples while no SiC peak observed in sputtered stoichiometric SiC film.

**Figure 5:**
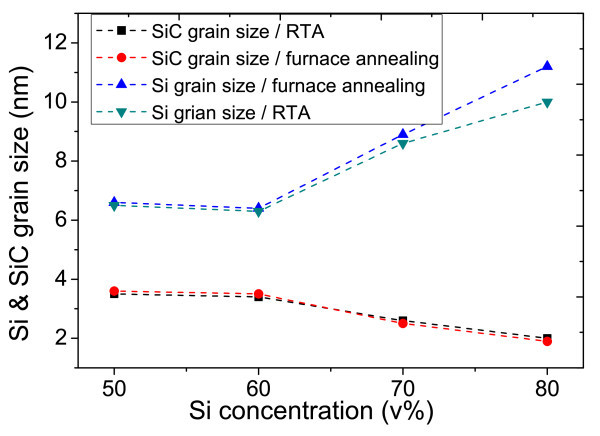
Si and SiC grain size from RTA and furnace annealing in different Si concentration.

The full width at half maximum (FWHM) of each XRD peak were carefully measured, and the nanocrystal size was calculated by Scherr formula,

(1) G=kλ/Δ(2θ)cosθ

where *λ *is the wavelength of the X-rays, *θ *is the Bragg diffraction angle at the peak position in degrees, *Δ*(2*θ*) is the FWHM in radian, and *k *is a correction factor. The value of *k *is usually chosen to be 0.9 for Si films. Nanocrystal sizes from RTA and furnace annealing samples are calculated by this formula and are indicated and compared in Figure 5.

In both RTA and furnace annealing samples, we can see that when Si concentration increases, Si grain size which is calculated from formula (1) also tends to increase. But the change is not significant until the Si concentration reaches 60 v% and grain size in furnace annealing samples tends to increase faster in high Si concentration (>70 v%). The same trend can also be observed in SiC-NC, the grain size of SiC crystal start to decrease when Si concentration falls below 60 v%.

**Figure 6:**
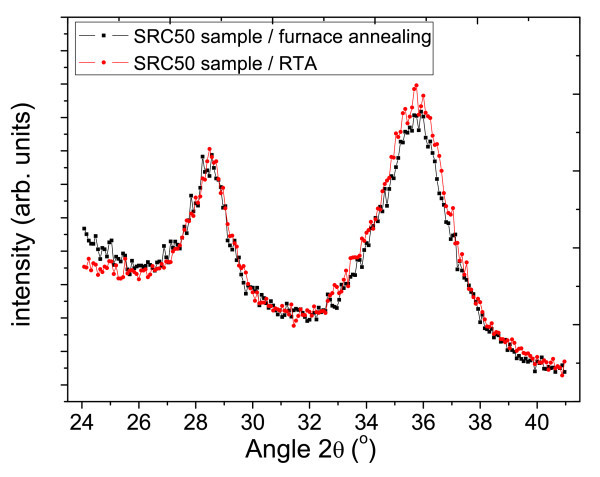
XRD curve comparison of SRC50 sample by RTA and furnace annealing.

**Figure 7:**
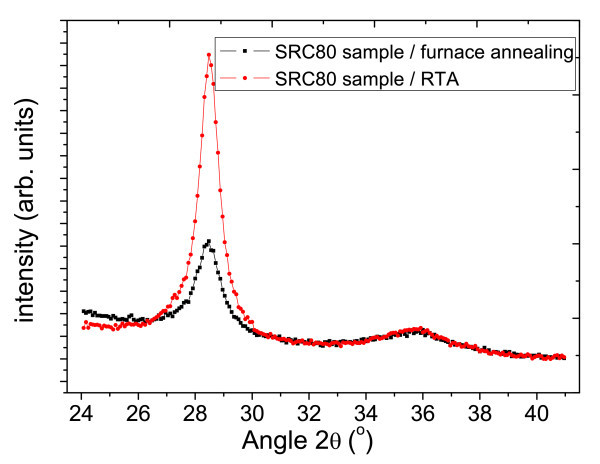
XRD curve comparison of SRC80 sample by RTA and furnace annealing.

The degree of Si crystallization can be estimated by the relative intensity of XRD peaks [15]. Figures 6 and 7 compare the RTA and the furnace annealing samples in different concentration. The relative intensity of two Si peaks (at 28.4°) is almost the same under low Si concentration at 50 v% (Figure 6). The intensity difference changes significantly when Si concentration increased to 80 v% (Figure 7). However, the difference of SiC peak intensity barely changes in both Si concentrations.

**Figure 8:**
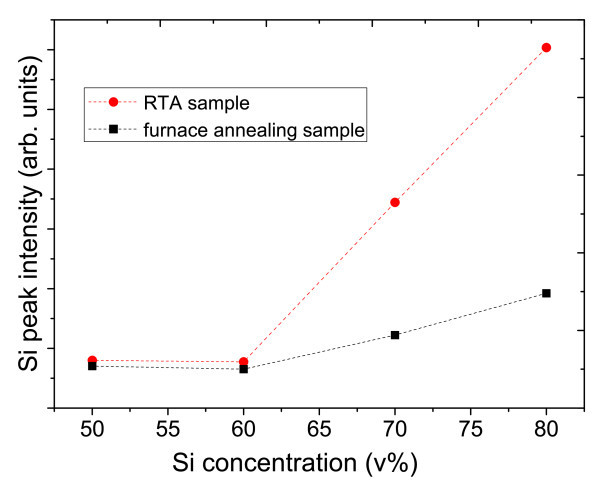
Si peak intensity of different Si concentration by RTA and furnace annealing.

We then further measure the intensity of Si peak from XRD result carefully as shown in Figure 8. Under low Si concentration range (50 and 60 v%), Si peak intensity of samples annealed by either RTA or furnace are almost the same. The intensity of RTA samples increased dramatically to two to three times higher compared to the furnace annealing samples when Si concentration increased above 60 v%.

### Raman investigation

**Figure 9:**
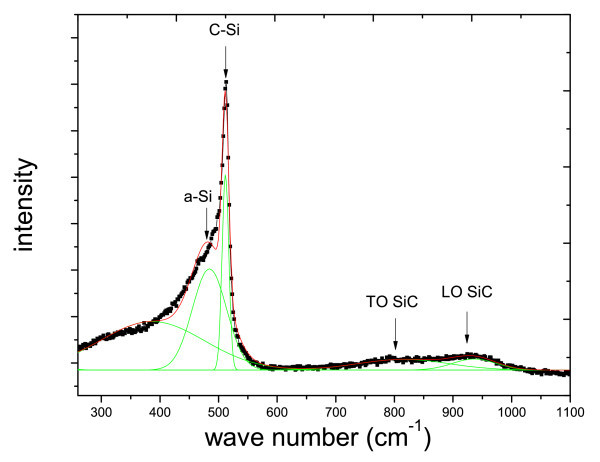
Raman spectrum of SRC60 after furnace annealing.

Figure 9 shows Raman spectrum of furnace annealed SRC60 sample. As we can see, the peak within the range of 400 to 600 cm^-^^1^can be de-convoluted to two main components: the peak centred at approximately 511 cm^-^^1 ^corresponds to Si nanocrystal phase and the peak centred at approximately 480 cm^-^^1 ^corresponds to the amorphous Si phase [6]. The hump at 400 cm^-^^1 ^may be assigned as partial breakdown of Raman selection rules [16]. Meanwhile, two small SiC peaks are also observed at approximately 800 and 940 cm^-^^1 ^attributed to the TO and LO of cubic and hexagonal SiC poly types [17,18].

**Figure 10:**
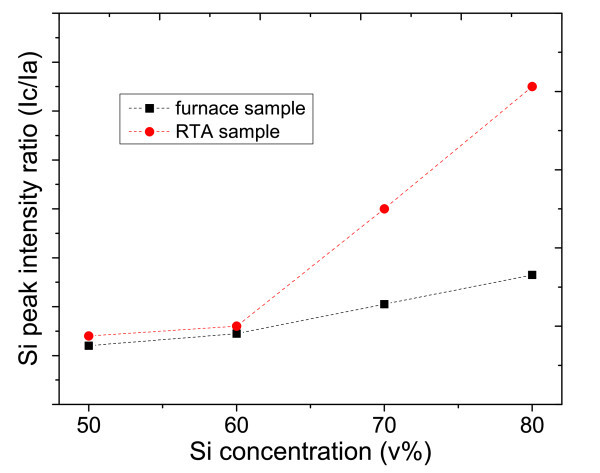
Calculated Si peak intensity ratios (*I*_C-Si_/*I*_a-Si_) in different Si concentration.

The degree of crystallization of Si nanocrystal could also be evaluated by calculating the intensity ratio of the crystalline Si peak and amorphous Si peak: *I*_C-Si_/*I*_a-Si _[6]. Figure 10 shows the relation of Si peak intensity ratio and silicon concentration in the SRC layers. The results indicate, for both RTA and furnace annealing conditions, when Si concentration increases, higher degree of silicon crystallization and less residual amorphous Si tend to be observed. Meanwhile, the samples from RTA show higher degree of Si crystallization in the matrix, comparing to the furnace annealing, especially in high Si concentration level.

### Discussion of structural difference and crystallization mechanism

RTA is considered as a positive annealing method in Si/SiC nanocrystalline system compared with furnace annealing. For the purpose of quantitative investigation, we calculate the degree of crystallization in all Si concentration range by comparing the RTA and furnace value ratio (*D*_RTA_/*D*_furance_) from the result of both XRD Si peak intensity (Figure 8) and Raman peak intensity ratio (Figure 10).

As shown in Table 3, from XRD analysis, the ratio remains at 1 when Si concentration is low (50-60 v%). The value comes to 2.4 under 70 v% Si concentrations and 2.8 under 80 v% Si concentrations. From Raman analysis, we can see the ratio stays also around 1 when in low Si concentration range (50-60 v%), and 2.2 in 70 v% Si concentration and 2.6 in 80 v% Si concentration.

**Table 3:** Degree of crystallization from RTA and furnace annealing in all Si concentration

	Si concentration (50 to approximately 60 v%)	Si Concentration (70 v%)	Si Concentration (80 v%)
++Degree of crystallization: *D*_RTA_/*D*_furance _(from XRD)	1	2.4	2.8
Degree of crystallization: *D*_RTA_/*D*_furnace _(from Raman)	1	2.2	2.6

The Si degree of crystallization ratio behaves in a similar overall increase trend from both XRD and Raman results. It's further confirmed that better Si nanocrystal crystallization could be obtained from RTA since more Si-NC are formed and less amorphous Si remained, especially under high Si concentration.

There are two possible crystal mechanisms to explain the main structural difference coming from RTA and furnace annealing procedure as we discussed above:

#### 1. Si-NC have not reached nucleation equilibrium in RTA

In classical theory of nucleation [19], free energy related to the formation of nanocrystal with radius *r *in an amorphous matrix can be described as:

(2) $$\Delta G_{\text{total}}=\text{4/3}\pi \;r^{\text{3}}\Delta G_{\text{phase}}+\text{4}\pi \;r^{\text{2}}\gamma$$

Here, Δ*G*_total _is the difference in free energy between the nanocrystal phase and the matrix phase, and *γ *is the interface energy, Δ*G*_phase _is the difference in free energy between the nanocrystal phase and the matrix phase. For negative Δ*G*_phase_, the critical nanocrystal size

(3) $$r^{\text{*}}=\frac{-\text{2}\gamma }{\Delta G_{\text{phase}}}$$

When *r *<r*, because of the decrease of the total free energy, NC tend to reduce in size and vanish in equilibrium. On the other hand, when *r *>r*, the NC must grow in size to reduce the total free energy until they reach equilibrium.

In our situation, obtaining reliable *γ *is extremely difficult, but J. K. Bording's group predicted the r* theoretically to be about 2 nm [20] for crystals and this value matches well with all our measured average SiC-NC size value in Figure 5. Basing on this theory, we may conclude, especially in high Si concentration, Si-NC may have not reached the equilibrium before the annealing temperature (1,100°C) drops in RTA. So, Si-NC whose grain size less is than 2 nm may have not completely vanished, thus more Si-NCs would be observed. The grain size increase trend in Figure 5 can further prove this point, we can see in high Si concentration region (70-80 v%) the Si grain size in RTA is smaller than furnace. This means Si-NCs in RTA could still grow up compare with samples of same Si concentration in furnace, which indicates Si-NC have not reached the equilibrium in RTA.

#### 2. Less SiC-NC pre-existed during ramping-up period before Si nanocrystal grow fast at high temperature

This explanation relies on the crystallization sequence. For both annealing techniques, the peak annealing temperatures (1,100°C) are the same, however the duration of temperature raise (from 500-1,100°C) is different. For the RTA system, it takes 45 s to increase but 40 min are needed to ramp up in furnace annealing situation. We believe the time period of temperature ramping up is crucial to Si crystallization process. From the result of Si degree of crystallization, much larger quantity of Si-NC are observed in RTA, which means Si-NC can be crystallized better in short ramping time situation. It may be because of the existence of SiC-NC before Si nanocrystal fast grows. As discussed earlier, Si nanocrystal start to form around 900°C, meanwhile, SiC-NC are induced to crystallize. Short ramping-up time in RTA may lead to less SiC nanocrystal before 1,100°C. As soon as the temperature rise up to Si fast crystallization point at 1,100°C, more Si-NC could be formed in RTA due to the decrease in SiC-NC.

## Conclusion

Si-rich SiC (SRC) layers with various Si concentrations were prepared by co-sputtering Si and SiC targets. Furnace annealing and RTA techniques were compared by studying the precipitation and crystallization of Si and SiC-NC with varying Si/SiC ratio after annealing.

Si and SiC-NC were observed by TEM in both furnace and RTA annealed at 1,100°C. SiC-NC are believed to be induced by Si nuclei from XRD spectra analysis. Meanwhile, when silicon concentration raised from 50 to 80 v%, increased size of Si nanocrystal (from 6 nm to 10 to approximately 12 nm) are observed but SiC nanocrystal size remains same (2 to approximately 4 nm).

Compared with furnace annealing, RTA samples reveal a better degree of crystallization on Si nanocrystal and less amorphous Si residual. More Si-NCs are detected by XRD and Raman analysis for this approach. This could possibly be explained by Si-NC not reaching nucleation equilibrium in the RTA or that less SiC-NC are present during the ramping-up period which increases Si-NC crystallization at high temperatures.

## Competing interests

The authors declare that they have no competing interests.

## Authors' contributions

ZW designed and carried out all the experiments as well as the article writing. SH produced all the TEM images. SH, MAG and GC all offered significant financial and technical support throughout the whole project.
